# Editorial: Towards 2030: Sustainable Development Goal 11: sustainable cities and communities. A sociological perspective

**DOI:** 10.3389/fsoc.2024.1428324

**Published:** 2024-06-06

**Authors:** Andrzej Klimczuk, Delali A. Dovie, Agnieszka Cieśla, Rubal Kanozia, Grzegorz Piotr Gawron, Piotr Toczyski

**Affiliations:** ^1^Department of Social Policy, Collegium of Socio-Economics, SGH Warsaw School of Economics, Warsaw, Poland; ^2^Centre for Ageing Studies, College of Humanities, University of Ghana, Accra, Ghana; ^3^Faculty of Geodesy and Cartography, Department of Spatial Management and Environmental Science, Warsaw University of Technology, Warsaw, Poland; ^4^Department of Mass Communication and Media Studies, School of Information and Communication Studies, Central University of Punjab, Bathinda, India; ^5^Institute of Sociology, Faculty of Social Sciences, University of Silesia in Katowice, Katowice, Poland; ^6^Media and Communication Department, Institute of Philosophy and Sociology, The Maria Grzegorzewska University, Warsaw, Poland

**Keywords:** sustainable cities and communities, smart homes, smart cities, SDG-11, green infrastructures, urban design, climate-resilient infrastructures

## Overview

This Research Topic addresses the eleventh Sustainable Development Goal (SDG), which is to “make cities and human settlements inclusive, safe, resilient and sustainable.” Several individual targets and indicators measure progress toward this goal. Researchers study, among others, urban inclusion, the influence of urban policy on socioeconomic disparities, and gentrification. This Research Topic primarily addresses the challenges and complexities of sustainable urban planning and development concerning decent work, economic growth, and associated crises due to their significant impact on urban living.

The presented selection of papers was edited in collaboration with the “*Frontiers in Sociology*” journal and includes five articles prepared in total by ten authors from the following countries: Albania, Canada, Italy, Portugal, and the United Kingdom. Three types of articles are included: one original research article (Guerra and Sousa), two review articles (Beretta and Bracchi; Sengupta and Sengupta), and two conceptual analyses (Ciampi and Sessa; Contini and Osmanaj). This Research Topic discusses themes covering social inclusion, neighborhood development, post-pandemic development, environmental justice, green cities and communities, climate-neutral cities and communities, smart cities and communities, and smart homes.

## Selected studies

The first study in the Research Topic by Guerra and Sousa, “*Dreaming is not enough. Audiovisual methodologies, social inclusion, and new forms of youth biopolitical resistance*,” focuses on the “not in education, employment, or training persons” (NEETs) that are among those most affected by specific social invisibility. Using an innovative “arts-based research” method and “youth-led participatory research,” called “The Neighborhood is Ours II!,” with young NEETs in the socially underprivileged Cerco neighborhood of Porto in Portugal in 2022, the study proposed a theoretical-empirical approach focused on visual/narrative sociology. They are, namely, using digital cinema, which was based on a short film about the narrative of a young NEET who used artistic practices to establish himself in the city of Porto as a cultural mediator. Essentially, the study shows how the arts promote social inclusion and minimize feelings of insecurity, in which the utilization of artistic practices plays a pivotal role in developing sustainable and alternative professional, social, and citizenship futures.

The subsequent two studies focus on the challenge of environmental justice. Beretta and Bracchi, in their paper “*Climate-neutral and smart cities: a critical review through the lens of environmental justice*,” argue that the fight against climate change can find a valid solution in technology and eco-innovations. This is evident in the growth strategies adopted, such as Europe 2020 and the European Green Deal, and in the primary research and innovation funding programs, such as Horizon Europe. In this context, the problem of environmental justice and the inclusiveness of the various initiatives implemented are attracting growing attention. The study results indicate that the strategic documents show that automatic participation translates into equality, while the guidelines show a more profound acknowledgment of the multidimensional nature of environmental justice. In addition, these entail distribution, rights, responsibilities, and recognition issues. Thus, the presented work is a preparatory and analytical tool that requires further definition and implementation of “climate city contracts” by the selected cities to assess how the issue of environmental justice is effectively being considered in specific contexts.

The topic of environmental justice is continued by Sengupta and Sengupta in the study “*SDG-11 and smart cities: contradictions and overlaps between social and environmental justice research agendas*.” This paper argues that information and communications technologies (ICTs) play a more significant role in achieving the SDGs. The authors specifically focus on SDG-11 and how cities increasingly incorporate ICTs to accomplish this goal. The study suggests that economic, social, and environmental benefits are possible with ICTs, even amid inequities in the distribution of environmental resources and services. The article combines a broad view of smart city environmental impacts with a deep examination of the intersection of social justice and environmental justice issues to create more holistic approaches for analyzing smart city projects' governance.

The team of Ciampi and Sessa presents a narrower perspective on smart solutions. Their essay, “*Pandemic and new perspectives on living: the role of the smart home*,” is based on ongoing multidisciplinary research. It offers theoretical and scenario considerations on the transformations of social rituals in housing contexts during the pandemic period. Their sociological perspective focuses on its usefulness in providing suitable tools to study the ambivalent and exceptional aspects to which living was exposed during the lockdown period and in the immediate aftermath. In this context, attention was paid to the phenomenon of the smart home as an “agent subject,” albeit inanimate, of the process of technological transformation of the housing unit. The study focuses on the evaluation of the smart home idea by considering the challenges of environmental and social sustainability.

The final paper included in this Research Topic is written by Contini and Osmanaj. The article is premised on the assumption that classical (Renaissance) humanism, understood as a philosophical and cultural trend, promoted science, art, literature, and ethics based on ancient values and codes of conduct through its focus on human with one's dignity and intellectual and moral abilities. These assumptions are still reflected in the incentives for social and civic activity and how selected cities are designed. However, considering the adverse effects of modern hyper-individualism, the authors propose a sociological discussion on the concept of a new humanism, which would arouse ethics of solidarity, recognition, and mutual respect in societies while focusing on protecting human integrity and support for sustainable development.

## Conclusion

The research results presented in this Research Topic of articles allowed the formulation of at least four directions for further research. These are: (1) digital divide in the context of smart and sustainable cities and communities; (2) governance, public management, and organizational management-related issues, including indicators for monitoring and evaluation of the multi-level, multi-stakeholder, and multi-sectoral approaches to environments, cities, and communities (see Stratigea et al., 2019); (3) studies that combine future cities and communities paradigms with concepts such as the silver economy, longevity economy, social economy, circular economy, green economy, and sharing economy (see Ciacci and Ivaldi, 2023); and (4) the ethics of artificial intelligence implementation for the smart and sustainable cities and communities (see Mazzi and Floridi, 2023; Son et al., 2023).

## Author contributions

AK: Conceptualization, Investigation, Project administration, Resources, Supervision, Writing – original draft, Writing – review & editing. DD: Conceptualization, Formal analysis, Writing – original draft, Writing – review & editing. AC: Conceptualization, Formal analysis, Writing – review & editing. RK: Conceptualization, Formal analysis, Writing – review & editing. GG: Conceptualization, Formal analysis, Writing – review & editing. PT: Conceptualization, Formal analysis, Writing – review & editing.
